# The Effect of Emotion and Reward Contingencies on Relational Memory in Major Depression: An Eye-Movement Study with Follow-Up

**DOI:** 10.3389/fpsyg.2016.01849

**Published:** 2016-11-22

**Authors:** Viola L. Nemeth, Gergo Csete, Gergely Drotos, Nora Greminger, Zoltan Janka, Laszlo Vecsei, Anita Must

**Affiliations:** ^1^Department of Neurology, Albert Szent-Györgyi Health Center, Faculty of Medicine, University of SzegedSzeged, Hungary; ^2^Department of Anaesthesiology and Intensive Therapy, Albert Szent-Györgyi Health Center, Faculty of Medicine, University of SzegedSzeged, Hungary; ^3^Neuroimaging Research Group, Research Center for Natural Sciences, Hungarian Academy of SciencesBudapest, Hungary; ^4^Department of Psychiatry, Albert Szent-Györgyi Health Center, Faculty of Medicine, University of SzegedSzeged, Hungary; ^5^MTA-SZTE Neuroscience Research GroupSzeged, Hungary

**Keywords:** major depression, relational memory, eye-tracking, facial emotion, virtual monetary reward and loss

## Abstract

**Background:** Episodic memory disturbances were found to constitute a potential trait marker for major depression (MD). The recall of positive or rewarding information in a relational context is specifically impaired. Eye-movement recording constitutes a novel, direct approach to examine implicit memory performance. Here we aimed to assess the effect of emotional context and implicit virtual monetary reward or loss on viewing patterns in association with relational memory in a 6-months follow-up study in MD.

**Materials and Methods:** Twenty-eight patients with MD and 30 healthy participants were trained to associate a face (happy/sad/neutral) with a background scene. After each pair a virtual monetary reward or loss appeared briefly. During testing, scenes were presented as a cue and then overlaid with three previously studied faces. Participants were asked to recall the matching face if present (Match trials), with eye-movements and subsequent forced-choice recognition being recorded.

**Results:** Explicit recognition of the matching face was impaired in the MD group as compared to controls. In correlation with this, viewing of the matching face was significantly reduced in the MD group. We found a significant interaction of group (MD vs HC) with the relational memory condition (Match and Non-match), facial emotion and monetary reward and loss. MD patients attended longer to previously rewarded stimuli, but significantly less to sad faces in the Match condition. The relational memory impairment persisted at follow-up and correlated with symptom severity both at baseline and follow-up. Viewing patterns associated with previous virtual reward were associated with clinical symptoms at follow-up.

**Conclusion:** Our current results provide novel evidence for a specific relational memory impairment in MD as supported by abnormal eye-movement behavior and a deficit in explicit recognition. MD patients showed an attentional bias to rewarded stimuli and decreased viewing of sad faces when relational memory information was present.

## Introduction

Primarily considered and classified as a mood disorder, changes in emotion are universally recognized as being inherent to major depression (MD) (APA, 2013). However, the way we feel and the way we process these emotions greatly interacts with cognitive aspects, i.e., the way we perceive and know the world around us (Dolcos et al., 2011). Research in the past few decades has focused on the significant impairment in cognitive function in MD patients. It is now becoming evident, that cognitive disturbances are not merely a consequence of symptoms of affect (Hammar and Ardal, 2009). Moreover, the cognitive impairment has become a relevant dimension of most psychiatric disorders, an aspect seriously affecting real-world functioning (Millan et al., 2012).

Cognitive deficits have widely been reported in MD, e.g., working memory and decision-making impairment in unmedicated MD patients, executive dysfunction in young adults with MD (Taylor Tavares et al., 2007; Castaneda et al., 2008). Various aspects of cognitive disturbance have been reported in the acute phase of the illness, e.g., executive dysfunction, including updating, shifting and inhibition processes (Harvey et al., 2004; Rogers et al., 2004). Cognitive deficits, e.g., mood-congruent memory retrieval impairment, has also been described in untreated, mild depression (Li et al., 2016b). Findings also indicate that an improvement in the cognitive status is not always in accordance with the remission of a depressive episode (Kennedy et al., 2007). Nevertheless, the cognitive deficit plays a crucial role in functional recovery from depression, whereas a persistent cognitive impairment might be an important factor associated with long-lasting disability in everyday functioning (Jaeger et al., 2006).

Among the various cognitive aspects associated with MD, memory disturbances have gained growing interest. Based on the emerging evidence of smaller hippocampal volumes, MD has become a potential risk factor for poor clinical outcome and consequent Alzheimer’s disease (MacQueen and Frodl, 2011). The smaller hippocampal volumes and metabolic changes in MD have been specifically related to episodic memory dysfunction (Mervaala et al., 2000), since episodic memory mechanisms are believed to be supported by the hippocampus (Cohen and Eichenbaum, 1993; Eichenbaum et al., 1994; Althoff and Cohen, 1999; Bird and Burgess, 2008; Konkel and Cohen, 2009). Episodic memory requires binding of an item to a particular context. This aspect of memory is most directly assessed with tests of associative or relational memory. Research evidence reported mild to moderate episodic memory impairments in MD even proposing episodic memory performance as a potential pre-morbid marker of depression (Airaksinen et al., 2004, 2007). Strikingly, the remission of depressive symptoms was typically not accompanied by improved episodic memory performance (Bierman et al., 2005; Airaksinen et al., 2006).

Good evidence has been reported that patients with MD have increased difficulty to exclude negative information – even if irrelevant – while performing a memory task (Levens and Gotlib, 2009). Complementary, the recall of positive or rewarding information is also impaired (Brittlebank et al., 1993; Levens and Gotlib, 2009). Patients suffering from MD might exhibit disadvantageous behavioral responses to reward or loss/punishment (Must et al., 2006; Eshel and Roiser, 2010). However, we found MD patients to be influenced by immediate large reward in a decision-making task, with reward having a greater influence on related response patterns (Must et al., 2006). More depressive symptoms have been related to perseveration in selecting options that led to overall gains (Byrne et al., 2016). Moreover, impairment in reward learning ability and in the modulation of behavior as a function of reward increased the risk for MD to persist after 8 weeks of adequate treatment (Vrieze et al., 2013). These results indicate that reward might have a more complex and implicit effect on cognitive function in MD.

A recent study by Wimmer and Shohamy (2012) has extended the role of the hippocampus beyond its role in associative memory formation to the ability to transfer and spread value between items. It has been suggested that the hippocampus contributes to an automatic assessment of value and to decision-making processes not necessarily driven by conscious awareness. Here we aimed to examine recognition of previously learned stimulus associations of scenes and emotional expressions (happy/sad/neutral faces) and manipulate the memory effects by virtual monetary reward or loss. We intended to establish a memory association between the scenes and facial emotional expressions and maintain the influence of the virtual monetary reward or loss more implicit. We performed eye-movement recording and explicit relational memory testing. Eye-movements are able to capture immediate access to stored information and may detect memory traces that do not even reach conscious stages, thus rapidly guiding to successful memory performance (Althoff and Cohen, 1999; Hannula et al., 2007; Hannula and Ranganath, 2009). Above this, eye-movements might be able to add insight into processes found to be altered when investigating reaction time differences in the context of emotional stimuli in MD (Naudin et al., 2014). Overall, eye movement analysis has been proposed as a promising new avenue in MD (Carvalho et al., 2015).

Firstly, we aimed to demonstrate a relational memory deficit in MD as revealed by a viewing preference and supported by subsequent explicit forced-choice testing. We hypothesized both a reduced viewing of the matching face for MD patients, compared to healthy controls (HC), as well as an impaired explicit recognition of the matching face in the MD group. Secondly, we expected episodic memory performance in MD patients to be influenced by the emotional context and implicit reinforcement by reward or loss. We hypothesized that mood-congruent biases on relational memory would be the most straight-forward expectations, involving a viewing preference for sad faces and stimuli associated with virtual monetary loss. We further assumed that facial emotion and virtual monetary reward or loss will have an interacting effect on relational memory performance. We expected that the implicit and explicit episodic memory performance would correlate with symptom severity in the MD group. Finally, a follow-up examination was performed 6 months after the first testing, aiming to examine whether specific aspects of the relational memory impairments in MD would potentially predict the patients’ clinical outcome.

## Materials and Methods

### Participants

Written informed consent was obtained from 28 patients (age: 49.22 ± 10.88 years) diagnosed with MD according to DSM-IV criteria and 30 healthy controls (HC, age: 46.83 ± 10.85 years) after approval of the study protocol by the local Ethics Committee (Ref. no.: 49/3-11/20144k). Patients were recruited from the Department of Psychiatry, University of Szeged, Szeged, Hungary. Patients were interviewed based on the SCID-I (APA, 2000) and were diagnosed by trained clinical professionals. All patients with a neurological illness, severe head injury, current axis I psychiatric disorder apart from MD or history of drug or alcohol dependence were excluded. HC participants were required to have no history of any psychiatric or neurological disorder. Two control participants reported to have taken antidepressants currently and were excluded therefore. Groups were closely matched for age, gender and years of education. All subjects had normal or corrected-to-normal (20/20) visual acuity (**Table 1**).

**Table 1:** Demographic and clinical characteristics.

	*N*	MD	HC	Statistics
		Mean *(SD)*	Mean *(SD)*	
Age	58	49.22 (10.88)	46.83 (10.85)	*t* = 0.723, *p* = 0.474
Gender (male/female)	58	5/23	10/18	χ = 2.276, *p* = 0.131
Years of education	58	12.15 (2.77)	13.61 (2.73)	*t* = -1.753, *p* = 0.088
Weeks between baseline and follow-up	14	24.07	-	-
HDRS_baseline_	28	18.15 (5.21)	-	*t* = 4.082, *p* = 0.004^∗^
HDRS_follow-up_	14	8.00 (6.28)	-	
Beck_baseline_	28	6.00 (3.58)	-	*t* = 1.269, *p* = 0.245
Beck_follow-up_	14	3.13 (3.23)	-	

*HDRS _baseline_ and HDRS _follow-up_: Total scores in Hamilton Depression Rating Scale at baseline and follow-up measurement, Beck_baseline_ and Beck_follow-up_ = Total scores in shortened version of Beck Hopelessness Scale at baseline and follow-up measurement.*

*^∗^Significant at p < 0.05*

Clinical symptoms were assessed using the following tests: the semi-structured interview of the Hamilton Depression Rating Scale and the shortened version of Beck Hopelessness Scale (Beck et al., 1974; Perczel Forintos et al., 2013) were registered to measure the severity and characteristics of depressive symptoms, the Hypomania Checklist (Angst et al., 2005) was applied to exclude hypomania or mania, the General Assessment of Functioning scale (APA, 2000) was applied to operationalize the patient’s ability in everyday functioning, while the National Adult Reading Test (NART) (Nelson, 1982; Nelson and Wilson, 1991) was used for pre-morbid IQ estimation. Associative memory has been assessed through eye-movements measuring fixation duration as well as explicit testing.

### Experimental Paradigm

The eye-tracking paradigm used here to assess implicit associative memory was a modified version of the task used by Hannula et al. (2007) and Williams et al. (2010). In the original version participants viewed three consecutive, randomized study blocks composed of the same 36 face–scene pairs during the training phase. The test phase followed immediately after completion of training and included 12 trials, each consisting of three faces overlaid on one scene. On the six Match trials, one of the three faces had been paired with the scene during the study phase, whereas on the six Non-Match trials none of the faces had been paired with that scene during training.

In the current study, fixation duration and eye-movements were recorded with an iView X^TM^ Hi-Speed SMI eye-tracker on 500 Hz connected to a Windows-based personal computer (SensoMotoric Instruments, Teltow, Germany^1^). Stable and consistent position of participants’ head was assured with a chin rest (distance from display: 90 cm/approximately 36 inches). Stimuli were presented on a 17″ CRT monitor (refresh rate: 100 Hz) using the iView X Experimental software.

Two types of stimuli were presented: neutral background images (colored scenes, sized 1024 pixels × 768 pixels) and faces of three different emotions: happy, sad, and neutral (314 pixels × 384 pixels). One face stimulus appeared in only one emotional expression. Stimuli of faces consisted of 18 male and 18 female face images obtained from the NimStim database (Tottenham et al., 2009), balanced for type of emotional expressions.

**FIGURE 1:**
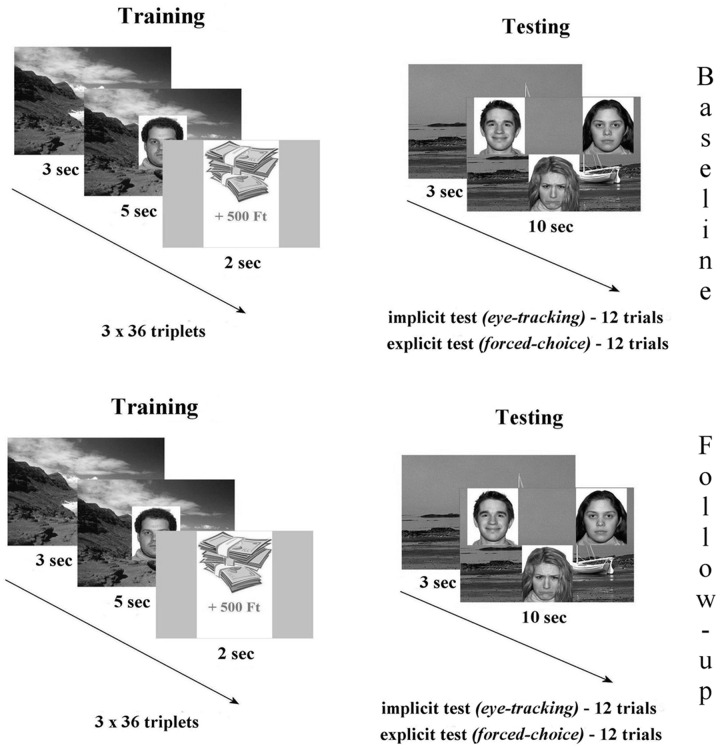
**The relational memory task was built of two major parts: (1) during the three consecutive training phases participants were asked to memorize a total of 36 pairs of backgrounds and facial emotional expressions (happy/sad/neutral).** After each pair a virtual monetary reward or loss appeared briefly, with no associated instruction provided. (2) During testing 12 background scenes were presented serving as the cue. Subsequently three faces of different emotions appeared overlaid on the background. Half (six trials) of the test trials contained the face previously paired with the cue (Match trials). For Non-match trials (six trials) none of the three faces was associated with the scene during training. Participants were asked to try and recall the matching face and keep viewing it (implicit testing). Explicit (behavioral) testing of relational memory by forced-choice recognition followed. In this phase, participants were instructed to press a button each rendered to the position of the face (upper left, upper right, and middle-bottom), or another button if neither face matched the scene. During the follow-up measurements, testing was performed using the same experimental paradigm entailing training, implicit and explicit memory examination.

The eye-tracking task was built of two major parts: (1) during the three consecutive training phases participants were asked to memorize a total of 36 pairs of backgrounds and facial emotional expressions (happy/sad/neutral). After each pair a virtual monetary reward or loss appeared briefly, with no associated instruction provided. (2) During testing 12 background scenes were presented serving as the cue. Subsequently three faces of different emotions appeared overlaid on the background. Half (six trials) of the test trials contained the face previously paired with the cue (Match trials). For Non-match trials (six trials) none of the three faces was associated with the scene during training. Participants were asked to try and recall the matching face and keep viewing it (implicit testing). Explicit (behavioral) testing of relational memory by forced-choice recognition followed. In this phase, participants were instructed to press a button each rendered to the position of the face (upper left, upper right, middle-bottom), or another button if neither face matched the scene. During the follow-up measurements, testing was performed using the same experimental paradigm entailing training, implicit and explicit memory examination (**Figure 1**).

### Stimulus Presentation

Eye-tracking procedure was applied under consistent lighting conditions with subjects sitting exactly 90 cm from the monitor, in a stable position throughout the task. SMI eye-tracker uses the registration of pupil and corneal reflection to calibrate the position of the eye by infrared camera. After a 9-point calibration all participants were asked to read the written instructions presented on the monitor. Sufficient time was provided for participants to raise questions as needed. All participants were instructed to try and memorize the background-face pairings for a subsequent recall.

In each of the three consecutive training phases, stimulus presentation started with a fixation cross for all 36 trials. Then a background scene appeared for 3 s, with an image of a facial expression being then overlaid on the background for 5 s. After each pair a virtual monetary reward of a smaller (500 Hungarian currency, HUF, approximately 1.6 Euro) or a larger amount (2000 HUF, approximately 6.5 Euro); or a virtual monetary loss of a smaller (500 Hungarian currency, HUF, approximately 1.6 Euro) or a larger amount (2000 HUF, approximately 6.5 Euro) was presented for 2 s. These fixed triplets were presented in a semi-random order during each training session. Participants were allowed to have breaks between the sessions.

During the “implicit” test phase a background scene appeared alone for 3 s with three faces being overlaid for another 10 s with eye-movements being recorded. Participants were instructed to search with their eyes and try to recall which of the three faces had been paired with the background scene during training, without giving an explicit response. Participants were asked to keep their eyes focused on the computer screen, even if no matching face was detected. On Match trials, the matching face was assigned equally often to the three display positions (i.e., upper left, upper right, and middle-bottom). Also, each emotional expression appeared equally often in each of the three different spatial locations (upper left, upper right, and middle-bottom position) Lists of stimuli were rotated and counterbalanced across participants to ensure that each scene was paired equally often with each face across the study. Virtual monetary reward or loss has also been rotated and counterbalanced across participants to ensure a balanced distribution of the four different monetary stimuli with the three different facial emotional expressions and sufficient power for subsequent statistical testing. It has been assured that each participant is exposed to different stimuli associations during the baseline and follow-up measurements to exclude any intrusion effects.

### Explicit Memory Testing

To assess explicit recognition of the face–scene pairings, we administered a subsequent four-alternative forced choice memory test after the implicit eye-movement phase was completed. Eye movements were no longer recorded in this phase. Participants viewed the 12 test displays in the same order and with the exact same background scene and face stimuli as during the preceding implicit test phase. Participants were asked to indicate the matching face by pressing a computer key corresponding to its position on the display or pressing the space bar if they thought none of the faces had been paired with that scene during training.

### Follow-Up Phase

Participants were invited to participate in a follow-up testing approximately 6 months after the initial, baseline measurements have been completed. Stimulus presentation has been performed similar to the initial testing. Clinical data has been recorded and the experimental paradigm has been administered in the same design, i.e., relational memory assessment with three training phases on the background – emotional facial expression – virtual monetary reward or loss stimulus triplets followed by the implicit testing phase with eye movement being recorded. The explicit memory assessment followed subsequently. Fourteen out of the 28 initial patients were available and agreed to participate in the follow-up phase.

### Statistical Analysis of Eye-Movement and Behavioral Data

Data analysis was performed oﬄine. Preprocessing of the data was carried out using software of the SMI eye-tracker and this was followed by statistical analysis.

We compared demographic parameters between the HC and MD group at baseline using independent samples *t*-tests. Clinical characteristics were addressed comparing the MD group at baseline and at follow-up using paired-sample *t*-tests.

Explicit memory performance was compared between groups with two-tailed, independent-samples *t*-tests. Results (*t*) were compared with the corresponding value of the Student’s distribution at the appropriate degree of freedom.

We assessed the effect of the different levels of virtual monetary reward and loss using repeated measures ANOVA, paired *post hoc* comparisons were Bonferroni corrected.

Group differences in overall viewing patterns were tested using a repeated measures analysis of variance (ANOVA) for fixation duration including relational memory condition (Match and Non-match), facial emotion type (happy, sad, and neutral) and virtual monetary effect (reward and loss) as within-subject factors and group (HC and MD) as between-subject factor. Interactions between conditions were further analyzed using paired-sample *t*-tests, and Bonferroni *post hoc* testing was used to assess the effect of facial emotion.

Pearson correlation was used to assess an association between viewing duration on the correctly matched face during Match trials and explicit memory performance. In order to perform this analysis, we first extracted viewing duration on the matching face for all Match trials which where subsequently explicitly identified as correct and correlated the obtained fixation times with explicit memory performance. This analysis aimed to detect the relationship between the two approaches of relational memory investigation.

We performed repeated-measures ANOVA to compare fixation duration at baseline and follow-up for MD patients for the relational memory condition (Match and Non-match), emotional expression (happy, sad, and neutral) and monetary reward or loss.

A potential association of viewing duration on the correctly matched face during Match trials as an implicit measure of relational memory, as well as between explicit behavioral performance and clinical data was assessed with Pearson correlation. Results were considered significant if the type I error remained below 0.05.

## Results

### Demographic and Clinical Characteristics

The MD and HC groups were closely matched for age, gender, and level of education (**Table 1**). Total HDRS scores at follow-up revealed a significant improvement in depressive symptom severity as compared to the first measurement (*t* = 4.082, *p* ≤ 0.004). Scores on Beck Hopelessness Scale also decreased, but the difference from baseline scores remained below statistical significance (*t* = 1.269, *p* = 0.245). All MD patients were treated with antidepressive medication and remained on the same schedule during the follow-up period.

### Baseline Testing

#### Explicit Memory Testing (MD > HC)

**FIGURE 2:**
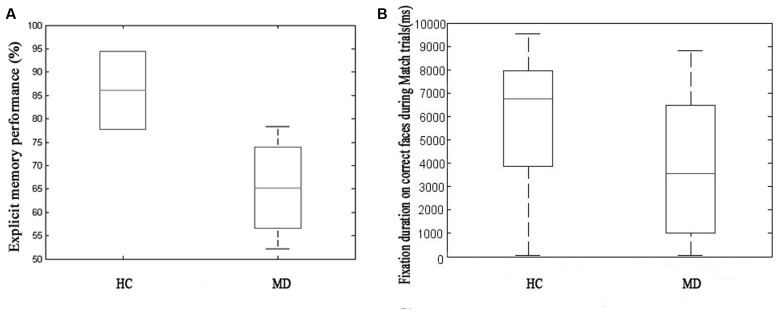
**Explicit memory performance during behavioral (forced choice button-press) testing on the left side of the figure (A)** and fixation duration on faces correctly identified as a Match on the right **(B)** comparing the healthy control (HC) and major depression (MD) group. On the explicit memory task MD patients performed on a significantly lower level compared to healthy control (HCs) (the HC group showed low variability, *M*_MD_ = 56.743% ± 27.865, *M*_HC_ = 78.205% ± 16.707, *p* ≤ 0.001). We found MD patients to fixate for a significantly shorter duration on correct faces (i.e., subsequently correctly identified as a matching face) during Match trials which contained relational memory information (*M*_HC_ = 5741.60 ± 2893.50, *M*_MD_ = 3795.30 ± 2899.00, *t* = 4.711, *p* ≤ 0.001) as compared to the HC group. The two different approaches to measure relational memory performance correlated with each other.

On the explicit memory task MD patients performed on a significantly lower level compared to HCs (*M*_MD_ = 56.743% ± 27.865, *M*_HC_ = 78.205% ± 16.707, *p* ≤ 0.001) (**Figure 2A**).

#### Implicit Memory Testing, the Effect of Facial Emotion and the Level of Monetary Reward or Loss (MD > HC)

We found MD patients to fixate for a significantly shorter duration on correct faces during Match trials (*M*_HC_ = 5741.60 ± 2893.50, *M*_MD_ = 3795.30 ± 2899.00, *t* = 4.711, *p* ≤ 0.001) as compared to HC (**Figure 2B**).

Performance on implicit (eye-tracking) and explicit (forced-choice button-press) testing correlated significantly with each other (*R* = 0.586, *p* ≤ 0.003).

**FIGURE 3:**
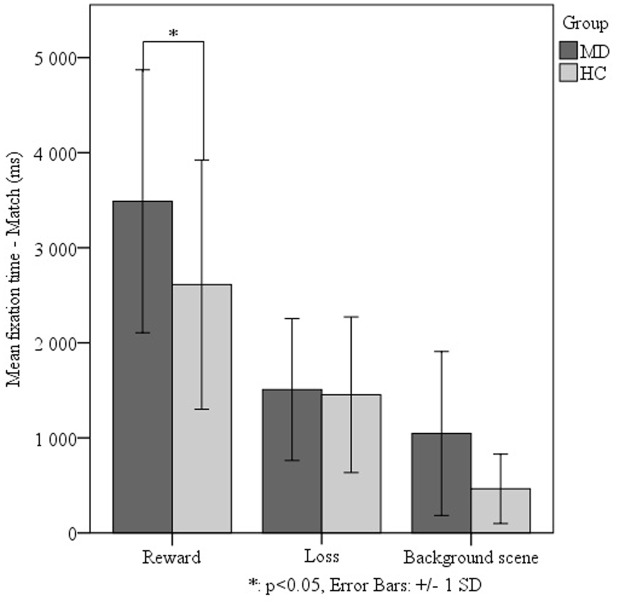
**Effect of virtual monetary reward or loss on fixation durations comparing the MD and HC groups.** In the Match condition – when relational memory information was present – we found MD patients to view faces associated with monetary reward for a significantly longer duration (*M* = 3489.05 ± 1382.788) than the HC group (*t*_48_ = 2.501, *p* = 0.016). This analysis was justified by higher order statistical analysis examining main effects of facial emotion and virtual reward or loss on relational memory performance.

**FIGURE 4:**
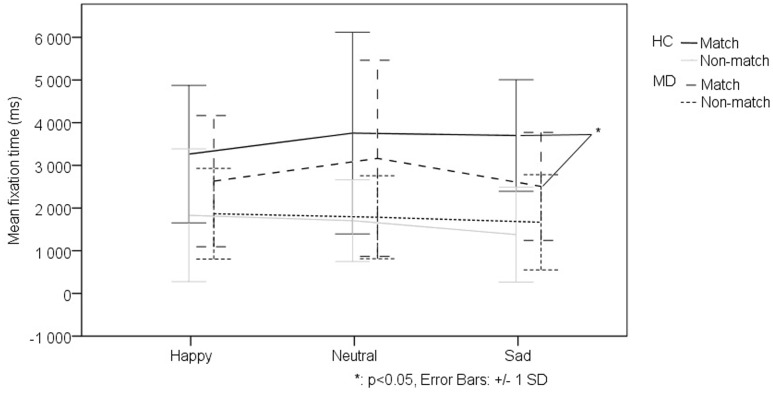
**Effect of facial emotion on viewing patterns comparing the MD and HC groups in trials associated with virtual monetary reward.** MD patients fixated on the sad match faces associated with monetary reward for a significantly shorter duration (*M* = 2291.124 ± 1304.498) than the HC group (*t*_48_ = -3,637, *p* = 0,001).

We investigated the effect of the different levels of virtual monetary reward or loss on the performance of the two groups. The repeated measures ANOVA did not detect a significant effect, thus we decided to analyze the two levels of reward and loss jointly. The repeated measures ANOVA of fixation duration revealed a significant main effect of relational memory condition (Match vs. Non-match) (*F*_1-38_ = 23.728, *p* < 0,001), a significant main effect of facial emotion (*F*_2-76_ = 7.287, *p* = 0,001) as well as a significant main effect of monetary reward or loss (*F*_1-38_ = 42.705, *p* < 0,001). Importantly, we found a significant interaction of group (MD vs. HC) by relational memory condition (Match and Non-match) by facial emotion (happy, sad, and neutral) by monetary reward and loss (*F*_2-76_ = 3.131 *p* = 0,049). The interaction testing of all factors revealed that, in the Match condition, the MD group viewed faces associated with monetary reward for a significantly longer duration (*M* = 3489.05 ± 1382.788) than the HC group (*t*_48_ = 2.501, *p* = 0.016) (**Figure 3**). However, the MD patients fixated on the sad match faces associated with monetary reward for a significantly shorter duration (*M* = 2291.124 ± 1304.498) than the HC group (*t*_48_ = -3,637, *p* = 0,001) (**Figure 4**).

#### Correlation of Implicit and Explicit Memory Performance with Clinical Symptom Severity (MD Only)

Performance on both explicit and implicit memory testing correlated negatively with depressive symptom severity as measured by clinical rating scales (explicit measure: HDRS_baseline_: *R* = -0.501, *p* ≤ 0.001, Beck_baseline_: *R* = -0.515, *p* ≤ 0.001, implicit measure: HDRS_baseline_: *R* = -0.311, *p* ≤ 0.022, Beck_baseline_: *R* = -0.280, *p* ≤ 0.040). This implies an association between better relational memory performance and lower scores on clinical rating scales, i.e., less severe symptomatology.

### Baseline > Follow-Up Testing

#### Explicit and Implicit Memory Testing

Explicit memory performance of the MD follow-up group did not improve significantly.

No significant follow-up effect in viewing durations could be detected when comparing baseline and follow-up measurements in the MD group in a repeated measures ANOVA design including relational memory condition (Match and Non-match), emotional expression (happy, sad, and neutral) and monetary reward or loss. Nevertheless, we found a correlation between performance of the MD follow-up group on implicit and explicit memory testing (*R* = 0.749, *p* ≤ 0.02).

#### Correlation of Implicit and Explicit Memory Performance with Clinical Symptom Severity (MD Only)

We found a negative correlation between severity of depressive symptoms based on HDRS scores and memory performance of the MD group at follow-up (explicit testing: *R* = -0.465, *p* ≤ 0.045, implicit testing: *R* = -0.428, *p* ≤ 0.067). However, we did not find a significant correlation with Beck scores (*p* > 0.05).

#### Correlation of Significant Differences in Viewing Patterns (MD > HC) at Baseline with MD Clinical Symptoms at Follow-Up

While implicit and explicit memory performance at baseline showed no correlations with symptom severity at follow-up (*p* > 0.05), we found that fixation duration on rewarded faces in Match trials at baseline correlated negatively with symptom severity at follow-up based on total HDRS scores (*R* = -0.399, *p* ≤ 0.016). However, no significant correlation with Beck scores was found (*p* > 0.05).

## Discussion

The current study provides novel evidence for a relational memory deficit in MD. We were able to demonstrate this deficit by studying eye-movements as an indirect measure of relational memory and by supporting our findings with explicit, forced-choice recognition of the previously associated stimulus pairs. Based on our results a deficit in retrieval of relational representations may well be presumed in MD as indicated by a significantly impaired match face recall during explicit testing accompanied by significantly shorter viewing durations on the matching face during eye-tracking. Episodic memory disturbances have been proposed as a potential pre-morbid marker of depression (Airaksinen et al., 2004, 2007). Here we aimed to investigate relational memory performance in MD as indicated by eye-movements in relation with explicit recognition measures. We used an approach found to be sensitive to relational memory deficits in patients with amnesia due to medial temporal lobe damage as well as schizophrenia. The relatively small number of test trials has to be noted as a limitation, although this paradigm is comparable to previous investigations of relational memory (Titone et al., 2004; Williams et al., 2010).

With our experimental design we aimed to detect the effect of facial emotion and monetary reward or loss on associative memory. However, we intended to separate the effect of virtual monetary reward or loss from social reward represented by faces with positive emotional valence or negative social stimuli, respectively, by not cueing former with the background scene, but merely establishing an implicit link between the facial emotion and the virtual monetary reward or loss. We hypothesized that a difference in interaction of these effects in the MD and HC group would suggest the possibility of an alternate neuronal processing of the effects of facial emotion and virtual monetary reward or loss on relational recall. Surprisingly, we found MD patients to fixate on stimuli associated with virtual monetary reward for a longer duration and the effect of emotional type also proved relevant. Fixation duration on sad faces associated with monetary reward was significantly decreased during Match conditions for the MD group. This suggests an emotional bias which interacts with the implied viewing preference for rewarded stimuli and potentially affects relational recall. However, the viewing preference for previously rewarded stimuli and the decreased fixation duration on sad facial expressions was rather unexpected.

Functional neuroimaging studies have suggested the role of an emotional or motivational pathway impairment in the dysfunctional reward-related processing in MD (Blood et al., 2010; Zhang et al., 2013). Reward learning mechanisms are known to be dependent on the striatum (Schultz, 2006). However, more recently, the ability to transfer value between stimuli thus biasing decisions not driven by conscious awareness has been attributed to the hippocampus (Wimmer and Shohamy, 2012). Here we aimed to manipulate associative encoding and retrieval by value representations and assess potential, more implicit effects on hippocampal mechanisms of action. Our analysis revealed that facial emotion and virtual monetary reward or loss had a significant effect on relational memory condition. We found the MD group to view faces previously associated with virtual monetary reward for a significantly longer duration during trials containing relational memory information, i.e., Match trials. Our previous results also indicated a greater influence of reward in MD (Must et al., 2006). This seeming contradiction might be explained by individual variations in neuronal activation patterns (Misaki et al., 2016), genetic variations and personality traits (Must et al., 2007; Byrne et al., 2016) or false recollection affecting the cognitive evaluation of rewarding stimuli (Davidson et al., 2002). Antidepressive therapy has also been found to have an enhancing effect on positive information processing (Wells et al., 2014). The neural background of an altered attentional focus on reward contingencies in depression presumably involves the fronto-striatal circuit. Converging evidence shows that depressed patients exhibit abnormal behavioral responses to reward contingencies corresponding to aberrant function in fronto-striatal systems (Eshel and Roiser, 2010). It has even been suggested that a disruption of this widely distributed network linked to a disturbance of the reward circuitry might serve as a biomarker for depression (Ma et al., 2012).

Previous studies have assessed emotional memory disturbances in MD and related disorders assuming that depression is associated with prolonged attention on negative information. Indeed, eye-movement studies revealed that the dysphoric group showed a significantly greater bias to maintain gaze longer on negative pictures, relative to control pictures with no evidence for an initial shift of orienting to negative cues (Caseras et al., 2007). Considering that depressed patients are characterized by reduced maintenance of gaze on positive stimuli and increased maintenance of gaze on dysphoric stimuli, one might presume that a maintained attentional preference also leads to a mood-congruent memory bias (Armstrong and Olatunji, 2012). However, results remained contradictory. Despite an impaired memory performance for emotional stimuli in depression and dysphoria, no mood-congruent memory bias could be identified for dysphoric or previously depressed patients (Williams et al., 1997; Yiend, 2010; Sears et al., 2011). Relational memory for negative emotional stimuli was found to activate the hippocampus and related areas both during encoding and retrieval. However, hippocampal activity and memory performance were not enhanced by negative emotionality (Onoda et al., 2009). Research results on the effect of emotional faces on attentional bias in MD further add to the complexity of interpretations. Symptom severity has been found to correlate both with an attentional bias for sad and happy faces (Duque and Vazquez, 2015). Some studies report a persisting attentional bias to sad faces in remitted MD (Soltani et al., 2015), while others found no significant difference as compared to controls concerning sad faces, but a decreased attentional bias toward happy faces (Li et al., 2016a).

The neuropathophysiology of depression also involves a limbic-thalamo-cortical circuit that includes the amygdala, striatum, medio-dorsal thalamus, and parts of the ventral and medial prefrontal cortex (Drevets, 2003). Reductions in hippocampal size and enlargement of the amygdala in MD were revealed as potential predictors of emotional memory function (Weniger et al., 2006). Increased activity of the fronto-limbic network and specifically amygdala involvement in episodic memory formation in first episode MD patients has been proposed as a neurocognitive trait or vulnerability factor for depression (van Eijndhoven et al., 2011). The degree of the over-recruitment of a neuronal network involved in emotional relational memory was also found to be related to the severity of clinical symptomatology (Hamilton and Gotlib, 2008). Strikingly, an eventual control of amygdala over-recruitment might serve as a novel therapeutic approach for the therapy of depressive symptoms (Young et al., 2014). Amygdala activation has reliably been found in response to both positive and aversive emotional stimuli (Ball et al., 2009). The role of amygdala has become evident in triggering responses and consequent decision-making processes to emotional stimuli, including facial emotions as well as monetary reward or loss – for a comprehensive review see (Gupta et al., 2011).

The episodic memory impairment previously described in MD has been found to persist after the remission of clinical symptomatology (Bierman et al., 2005; Airaksinen et al., 2006). Here we found preferential viewing of the matching face subsequently recognized as correct as well as behavioral measures of relational memory to correlate negatively with symptom severity. Thus, better relational memory performance implies less severe clinical symptomatology. However, implicit and explicit memory performance at follow-up did not improve significantly as compared to baseline. Again, preferential viewing of the matching face correlated positively with the explicit choice and negatively with symptom severity. Viewing patterns of MD patients were found to be similar to controls for Non-match displays, which contained no relational memory information. Converging research evidence supports the clinical notion that cognitive impairment may remain and persist residually in the remitted state of MD (Hasselbalch et al., 2011). Mood congruent information-processing biases also appear to have an important role as vulnerability factors for the development, maintenance (Everaert et al., 2015) and even recurrence of depression (Kellough et al., 2008; Gotlib and Joormann, 2010; Mannie et al., 2015). Specifically, persistent emotional memory impairment has been linked to distinct neural mechanisms mainly involving the frontal cortical pathways (van Wingen et al., 2010). Strikingly, impaired responsiveness to reward related to the fronto-striatal network has also been reported in remitted MD suggesting this to be a trait marker for depression (Dichter et al., 2012).

With our follow-up examination performed 6 months after the first testing, we aimed to examine whether specific aspects of the relational memory impairments in MD would potentially predict the patients’ clinical outcome. We did not reveal any significant follow-up effects, the relational memory deficit persisted. We found that longer fixation duration for faces associated with virtual reward in Match trials at baseline correlated with a less severe clinical symptomatology and a better outcome at follow-up. We remain critical and aim to further extend our investigations considering the low number of patients examined at follow-up. Here we also need to note that all patients were treated with antidepressive medication at the time of investigation. However, all patients remained on the same medication during the follow-up period. Previous research findings have suggested a distinct pattern of cognitive impairment involving memory aspects persisting beside antidepressive medication (Luo et al., 2013).

In sum, our results indicate a relational memory impairment in MD, as revealed by abnormal eye-movement behavior and a deficit in explicit recognition. We found a significant effect of facial emotion and virtual reward or loss on relational memory performance. Implicit, i.e., fixation duration, and explicit measures of relational memory correlated with each other and with clinical symptoms. We were not able to detect a significant improvement in relational memory performance at 6 months follow-up. Viewing patterns associated with reward at baseline in conditions when relational memory information was present suggested better clinical symptomatology and outcome. Therapeutic implications involving crucial cognitive aspects of the disorder (Trivedi and Greer, 2014) might consider emphasizing the role of reward contingencies related to the affective etiology of MD. Eye tracking might yield new insights into the assessment of cognitive function in MD (Armstrong and Olatunji, 2012; Carvalho et al., 2015).

## Author Contributions

VN has performed the cognitive and neuropsychological testing, contributed to data analysis and interpretation, as well as manuscript preparation and writing. GC has contributed significantly to data analysis and interpretation, as well as manuscript preparation and writing. GD has contributed significantly to preparation of cognitive and neuropsychological testing, programing as well as data analysis. NG has performed patient enrollment, clinical and neuropsychological testing and participant follow-up. ZJ has contributed to study planning and preparation, data interpretation and manuscript preparation. LV has participated in data interpretation, manuscript preparation and writing. AM has contributed to study planning and preparation, data collection and analysis, data interpretation, manuscript writing.

## Conflict of Interest Statement

The authors declare that the research was conducted in the absence of any commercial or financial relationships that could be construed as a potential conflict of interest.

## Footnotes

1 http://www.smivision.com/
